# Effects of temporally regular versus irregular distractors on goal-directed cognition and behavior

**DOI:** 10.1038/s41598-022-13211-3

**Published:** 2022-06-15

**Authors:** Troby Ka-Yan Lui, Malte Wöstmann

**Affiliations:** 1 Department of Psychology, University of Lübeck, Lübeck, Germany; 2 Center of Brain, Behavior, and Metabolism, University of Lübeck, Lübeck, Germany

**Keywords:** Psychology, Human behaviour, Attention

## Abstract

Human environments comprise plenty of task-irrelevant sensory inputs, which are potentially distracting. Auditory distractors often possess an inherent temporal structure. However, it is largely unknown whether and how the temporal regularity of distractors interferes with goal-directed cognitive processes, such as working memory. Here, we tested a total sample of N = 90 participants across four working memory tasks with sequences of temporally regular versus irregular distractors. Temporal irregularity was operationalized by a final tone onset time that violated an otherwise regular tone sequence (Experiment 1), by a sequence of tones with irregular onset-to-onset delays (Experiment 2), and by sequences of speech items with irregular onset-to-onset delays (Experiments 3 and 4). Across all experiments, temporal regularity of distractors did not modulate participants’ primary performance metric, that is, accuracy in recalling items from working memory. Instead, temporal regularity of distractors modulated secondary performance metrics: for regular versus irregular distractors, recall of the first item from memory was faster (Experiment 3) and the response bias was more conservative (Experiment 4). Taken together, the present results provide evidence that the temporal regularity of task-irrelevant input does not inevitably affect the precision of memory representations (reflected in the primary performance metric accuracy) but rather the response behavior (reflected in secondary performance metrics like response speed and bias). Our findings emphasize that a comprehensive understanding of auditory distraction requires that existing models of attention include often-neglected secondary performance metrics to understand how different features of auditory distraction reach awareness and impact cognition and behavior.

## Introduction

Sensory events in human environments often possess an inherent temporal structure (e.g., a ticking clock in the living room). Some of these events are relevant for goal-directed behavior, while others are irrelevant and potentially distracting. Dynamic attending theory^1^ suggests that temporal regularity of task-relevant stimuli guides attentional resources to the expected onsets of stimuli, and hence facilitates sensory processing at these time points. The prevalence of temporal regularity in task-irrelevant stimuli urges for the question of whether temporal regularity of distraction impacts humans’ execution of goal-directed cognitive operations.

Shielding memory representation against external distraction is important to successfully maintain relevant information^2,3^. The irrelevant-sound task^4,5^, in which participants need to maintain the order of target numbers in memory while ignoring auditory distractors, provides a gateway to probe how different psycho-acoustic features of distractors interfere with working memory. The irrelevant-sound effect refers to the observation that, compared with stationary background noise or silence, memory interference is larger for irrelevant sounds, such as speech distractors (e.g.^6–8^) or sequences of pure tones with changing frequencies (e.g.^9,10^), although the size of the irrelevant-sound effect is typically larger for speech versus tone distractors^10,11^. Of relevance to the present study, the degree of interference has also been shown to be modulated by the violation (e.g.^12^), or the lack of (e.g.^13^) repeating structure in the distractor sequence, which could be explained by the auditory deviant hypothesis^14,15^ or by the changing-state hypothesis^16,17^. Violation of the regular structure of distractor sequences may interfere with working memory by means of attentional capture, which refers to the orientation of attentional resources to a stimulus outside the current focus of attention when the stimulus deviates from expectation^12,18^. Alternatively, a changing-state distractor (e.g., A-C-D-J-E as opposed to A-A-A-A-A) is supposed to interfere with the order of target stimuli maintained in memory in the irrelevant-sound task^13,19^.

Given the rich evidence on how different features of distractors interfere with working memory, the paucity of studies on the role of temporal regularity of distractors is surprising. The current study aimed at filling this gap by investigating how the temporal regularity of distractor sequences influences goal-directed working memory. The onset time of distraction has recently been shown to modulate working memory interference^20,21^, demonstrating that temporal features of distractors may play an important role in the susceptibility to distraction in working memory tasks. When it comes to temporal regularity, temporally irregular distractors were recently found to be more disruptive to the detection of deviance in unmasked targets^22^. For concurrently presented target and distractor streams, this study supports the view that temporally regular versus irregular distractors differentially interfere with goal-directed cognitive operations. In studies using irrelevant-sound tasks, however, inconsistent results were found, such that participants either performed better^23^ or worse^24^ with regular versus irregular distractors. It is thus an unresolved question whether and how the temporal regularity of distractors during memory retention affects working memory performance.

Different theoretical accounts may give rise to different predicted effects regarding how the temporal regularity of distractors may disrupt working memory. Within the theoretical framework of the irrelevant-sound effect, previous studies explained the differential effect of isochronous versus random temporal structures on serial memory accuracy based on the changing-state hypothesis. Temporally regular distractor sequences may facilitate^23^ or interfere with^24^ the perceptual organization of distractors, thereby modulating the precision of the serial memory representation. However, previous evidence also showed that serial order memory depends on the position, rather than the timing, of targets^25,26^. Whether violation of the temporal regularity of distractors acts as a changing-state sequence and influences serial order memory thus remains unclear. Of note, the current study does not strictly test the changing-state hypothesis as the distractors used within each individual experiment were implemented either as a steady-state (in Experiments 1 and 2) or a changing-state (in Experiments 3 and 4) sequence.

Alternatively, according to the auditory deviant hypothesis, temporally regular distractors may facilitate the formation of an expectation regarding when the next distractor may occur. The deviation from temporal regularity may then potentially capture attention, rendering the distractors harder to ignore. Distractor sequences with temporal deviants interfere with the serial recall of concurrently presented to-be-remembered sequences^27^. Electrophysiological studies also revealed that the human auditory system detects changes in the temporal regularity of ignored stimuli^28^, which suggests the general potency of temporal (ir)regularity to capture attention.

More generally, the temporal regularity of distractors may facilitate or disrupt the shielding of working memory from distractions based on different theoretical considerations. First, some stimulus properties that typically facilitate auditory target processing (e.g., acoustic detail and voice familiarity) were shown to disrupt memory performance when occurring in the distractor^29,30^. As the temporal regularity of targets typically aids target processing^31,32^, temporally regular distractors may in turn be more distracting to the participants. Second, temporal expectation formed by regular temporal structures may decrease the degree of distraction. Previous evidence shows that participants performed better when they had foreknowledge about the deviation in distractors^33^, suggesting that expectation may reduce susceptibility to distraction.

The inconsistent evidence in the current body of literature may be due to different reasons, and the current study probed into each of the following conjectures, using a series of experiments. First, the temporal regularity effect may vary depending on the type of temporal (ir)regularity employed. As mentioned, the violation of temporal regularity embedded in the distractor sequence may also potentially capture attention. It is therefore important to test whether deviation from temporal regularity of distractors influences working memory performance. Experiment 1 in the current study investigated the deviant effect in time by manipulating the stimulus onset asynchrony of a final distractor tone in a distractor sequence. Experiment 2 manipulated temporal regularity by using isochronous (regular) versus random (irregular) temporal structure for all items in a distractor sequence.

Second, the modulatory effect of temporal regularity in the distractor sequences on working memory may depend on the type of sound events used in the irrelevant-sound task^34^. Studies on temporal regularity in distraction used distractors from a wide range of stimuli, spanning from pure tones^35^ to speech items^24^. It is possible that the temporal regularity effect is more prominent with distractors that are more difficult to ignore. Thus, we started out with pure tone distractors in Experiments 1 and 2 and found that their temporal (ir)regularity did not affect working memory recall accuracy. We then employed spoken numbers as distractors in Experiments 3 and 4 to increase the degree of distraction.

Third, previous studies focused largely on primary performance metrics like the accuracy of memory recall, which reflects the precision of memory representation. However, it is conceivable that temporally regular versus irregular distractors rather affect secondary performance metrics of response behavior during memory retrieval, which may involve metacognitive evaluation and threshold setting^36,37^. Metacognition, usually operationalized as confidence rating, refers to one’s evaluation and knowledge of the cognitive processes^38^. Metacognitive monitoring and control have been suggested to be involved in strategic regulation during memory recall^36^. Specifically, response bias may represent a threshold in memory recognition: a participant would only respond that they remember the item (i.e., “old” item) if their confidence is higher than a certain threshold, which is related to faster response time^39–41^. Therefore, studying how the temporal regularity of distractors influences response speed, confidence, and response bias would be required to obtain a comprehensive understanding of whether the temporal (ir)regularity of auditory distraction reaches awareness.

It is possible that, instead of interfering with the serial order memory as suggested by the changing state hypothesis, the temporal regularity of distractors may have a more general impact on the goal-directed response behavior. In such case, instead of directly interfering the serial memory recall accuracy, the temporal regularity of distractors may modulate the response behavior (e.g., response speed and bias) which are less reflective of the serial memory maintenance but still sensitive to how distractors affect goal-directed behavior^22,42^. Previous research found that temporal regularity of target stimuli increases confidence ratings, which was attributed to an increase in processing fluency, or the subjective experience of ease during information processing^43,44^. Furthermore, previous studies revealed a facilitatory effect of temporal regularity on response time in target detection tasks^31,32,45^, suggesting that the periodicity in regular stimuli may facilitate motor preparation. To explore whether temporal regularity of distractors also modulates processes other than the precision of items represented in memory, we included secondary performance metrics response time in Experiment 3, as well as response bias and confidence ratings in Experiment 4. In addition, we included visual distractors in Experiment 4 to investigate if the temporal regularity effect of distractors on working memory, if any, is modality-specific.

Across Experiment 1 to 4 in the current study, different variants of working memory tasks were used to investigate whether temporal regularity of distractor affects working memory. Overall, we found no temporal regularity effect on the primary performance metric memory recall accuracy. However, temporal regularity was found to modulate participants’ secondary performance metrics, reflected by response speed in Experiment 3, as well as response bias and confidence in Experiment 4.

## Methods

### Participants

Across all 4 experiments, N = 90 native German speakers (70 females, 20 males) aged 19 to 64 years (mean = 24.81, SD = 3.95) participated, among which N = 89 participants were included in the analyses. All participants provided written informed consent. Participation was compensated financially or by course credit. According to self-report, all participants were right-handed, had normal hearing, and had normal or corrected-to-normal vision. The experimental procedures were approved by the local ethics committee of the University of Lübeck and in accordance with the Declaration of Helsinki.

Due to technical issues, one participant in Experiment 1 finished only 184 out of 250 trials, the rhythmicity rating of one participant in Experiment 2 was not recorded, and the data of one participant in Experiment 3 were overwritten and thus missing. Two participants participated in more than one of our experiments that were at least 5 months apart from each other. Detailed information on participant samples for individual experiments can be found in Table 1.

**Table 1:** Details of experimental manipulations for each experiment.

		Experiment 1	Experiment 2	Experiment 3	Experiment 4
Participants	Sample size	n = 21 (14f, 7m)	n = 19 (18f, 1m)	n = 20 (16f, 4m)	n = 30 (22f, 8m)
	Age (years; M = mean; SD = standard deviation)	20–64 (M = 26.95, SD = 2.7)	19–38 (M = 24.8, SD = 5.74)	19–27 (M = 22.7, SD = 2.25)	19–32 (M = 24.7, SD = 3.54)
Encoding	Target duration	5.1 s	~ 6.6 s	~ 5.6 s	2 s
	# of targets	9	9	8	8
Retention	Retention duration	3 s	5 s	8 s	8 s
	Distractor onset delay	0.5–1 s	1.035–1.835 s	1.035–1.835 s	1.035–1.835 s
	Distractor type	Pure tones (1000 Hz)	Pure tones (440 Hz)	Spoken numbers	Spoken numbers/numbers on screen
	Distractor duration	1.675–1.925 s	1.8 s	~ 5.6 s	~ 5.6 s
	Factors (# levels)	SOA (5)	Regularity (2)	Regularity (2)	Regularity (2) × Modality (2)
Retrieval	Response device	Keyboard	Mouse	Mouse	Response pad
	Outcome measure	Accuracy	Accuracy	Accuracy, Speed (1/RT)	Accuracy, Speed (1/RT), Criterion, Confidence
	Number of trials	250 (50 per condition)	108 (54 per condition)	120 (60 per condition)	256 (64 per condition)
	Number of blocks	5	2	2	4
	Inter-trial interval	1 s	1 s	1 s	0.73–4 s
	Block design?	No	No	No	Modality (visual/auditory)
Apparatus	Lab/online	Lab study	Online study	Lab study	Lab study
	Sound presentation	Headphone (Sennheiser HD 280 Pro)	Headphone (n = 10), speakers (n = 9)	Headphone (Sennheiser HD 280 Pro)	Headphone (Sennheiser HD 280 Pro)

*m* male, *f* female, # number, *n* sample size.

### Stimuli and Procedure

Inspired by the well-established irrelevant-sound paradigm^4^, we used serial working memory tasks (Experiment 1–3) and a recognition memory task (Experiment 4) to present temporally regular versus irregular distractors during memory retention. For all experiments, each trial consisted of a memory encoding, retention, and retrieval phase. Prior to the execution of the main task, participants were instructed to maintain the order (Experiment 1 to 3) or the position (Experiment 4) of the target stimuli in mind while ignoring the distractor sequence presented during the retention period. The distractor onset delay, i.e., the onset of the distractor sequence after the offset of the target stimuli, varied across trials (see “Distractor onset delay” in Table 1). Here, we describe the general experimental design and important manipulations for each experiment (see Table 1 for all details).

**Figure 1:**
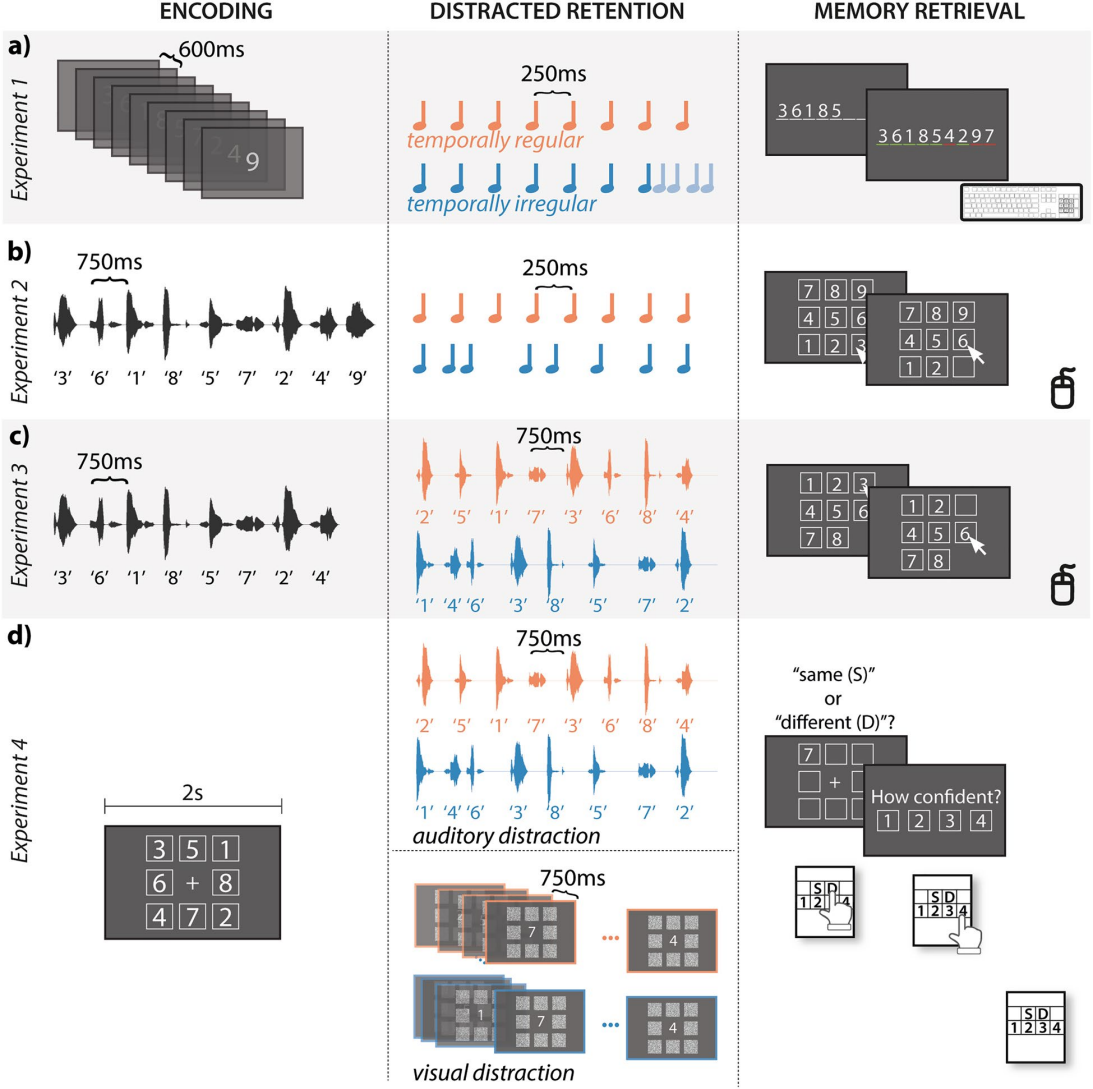
In all four experiments (**a**–**d**), participants maintained the target numbers in memory during the retention period while ignoring the distractors (blue/orange). In Experiment 1 (**a**), temporal regularity of distractors was manipulated by altering the onset of the last distractor tone such that it was either identical (regular, orange), or shorter/longer (irregular, blue) than the stimulus onset asynchrony of the preceding distractor sequence. In Experiments 2 to 4 (**b**–**d**), temporal regularity of the distractors was operationalized by isochronous (regular, orange) or irregular (blue) temporal structure of the entire sequence of distractors. After the retention period, participants responded with a mouse to select the numbers in their order of presentation from a visually presented number pad (Experiments 2 and 3), with a number pad on a keyboard (Experiment 1), or with a response pad (Experiment 4).

In Experiment 1 (Fig. 1a), a target sequence with numbers from 1 to 9 was presented visually in the center of the screen, in a random order, during the encoding period. The duration of each number presentation was 300 ms, the stimulus onset asynchrony (SOA) between numbers was 600 ms, and the total duration of the target stimuli was 5100 ms. During the retention period, a distractor sequence was presented. The distractor sequence consisted of eight 1000-Hz pure tones with a 4-Hz presentation rate (i.e., SOA of 250 ms). The SOA of the last distractor tone was manipulated across 5 levels: 125 ms, 187.5 ms, 250 ms, 312.5 ms, and 375 ms. Regularity of the distractor sequence was given when the last distractor tone SOA was identical (i.e., 250 ms; Regular) versus different (i.e., 125, 187.5, 312.5, and 375 ms; Irregular) from the SOAs of the previous tones. After the retention period, participants recalled the target number sequence by typing it on the number pad of a keyboard. Afterwards, feedback was provided with green and red underscores under correct and incorrect answers, respectively.

In Experiment 2 (Fig. 1b), the target was a pseudo-random permutation of German utterances of the digits 1 to 9, pronounced by a female speaker, with the constraint that no succeeding numbers (e.g., 3 and 4) be presented consecutively. The average duration of the numbers was 595 ms and the SOA between numbers was 750 ms^29^. The distractor sequences consisted of eight 440-Hz pure tones (i.e., musical note A4) and were either temporally regular or irregular. For the regular sequence, the SOA was 250 ms (4 Hz). For irregular sequence, the SOA was randomly selected between 100 and 400 ms (10 ms steps), with the constraint that the average SOA, as well as the last SOA, were each 250 ms. In the retrieval period, participants navigated the screen with a mouse and selected the numbers from a number pad presented on the screen. No feedback was provided afterwards.

The target stimuli in Experiment 3 (Fig. 1c) consisted of 8 numbers (numbers 1 to 8) also in a pseudo-randomized order as in Experiment 2, spoken either by a female or a male speaker. The numbers were shortened to 350 ms using Praat (version 6.1.16; http://www.praat.org/). The perceptual center of each stimulus was determined by first creating the 15-Hz lowpass-filtered envelope of the stimulus using Hilbert transform, and then finding the time point where the envelope reached 50% of the peak of the first syllable^46,47^. The interval between the perceptual centers of the numbers was 750 ms. The temporal regularity of the distractor was manipulated in the same way as in Experiment 2, where spoken numbers of the distractor sequence were spoken by a female voice in case the target was spoken by a male voice and vice versa. For regular sequences, the SOA was 750 ms (1.33 Hz). For irregular sequences, the SOA was randomly selected between 400 and 1100 ms (10 ms steps), with the constraint that the average SOA, as well as the last SOA, were each 750 ms. Furthermore, a lower bound of temporal irregularity was implemented by the constraint that the standard deviation of SOAs within a trial was larger than 200 ms. As in Experiment 2, participants navigated on the screen with a mouse to select the numbers on the number pad. No feedback was provided also in Experiment 3.

In Experiment 4 (Fig. 1d), instead of having an acoustically presented number sequence, the target stimulus was a visually presented 3 × 3 matrix, including 8 numbers in 8 positions (the center of the matrix was empty during encoding). The target stimulus was presented for 2000 ms. The same parameters for the distractor stimuli as Experiment 3 were used in the auditory modality, except that the SOA for the irregular sequence was randomly selected using 16.7 ms steps to account for the refresh rate (60 Hz) of the monitor, such that the irregular sequences were comparable between auditory and visual modalities. In the visual modality, the number sequences were presented in the center of the screen consecutively with the same manipulation in terms of the temporal regularity of the distractors. After the retention period, a display with one probe number at one position was presented. Participants had to identify whether the probe number matched the target number in the encoding display at this position. The button assignment (i.e., left versus right) was counterbalanced for “match” versus “no match” responses across participants. Afterwards, participants indicated how confident they were that they answered correctly on a 4-point scale (1 = not confident at all, 4 = very confident). Participants also received no feedback in Experiment 4.

In addition to Experiments 1 to 4, a control experiment (N = 18, mean age = 23.68 years, SD = 2.83, 16 females, 2 males) was conducted to demonstrate the strength of interference by the tone and spoken number distractors, respectively, relative to when there was no distractor (i.e., quiet control condition). The target stimuli and response method were the same as in Experiment 3. The experiment was divided in four blocks with the lengths of the retention period fixed within each block. For half of the blocks, the retention period was 5 s as in Experiment 2; for the other half, the retention period was 8 s as in Experiment 3. In the 5 s retention blocks, either no distractor (quiet control; 50% of all trials) or a tone sequence (distractor presence; 50% of all trials) used in Experiment 2 was presented during the retention period. The tone sequence was either temporally regular (50% of the distractor presence trials) or irregular (50% of the distractor presence trials) to maintain the same context as in the main experiment. Trials with different temporally regular and irregular distractors were combined in the analysis. The manipulations were the same in the 8 s retention blocks, but with the spoken number sequences from Experiment 3 serving as distractors. There were 192 trials in total and 48 trials for each of four conditions [distractor (present vs absent) × retention period (5 s vs 8 s)]. Block order was counterbalanced between participants, with half of the participants starting the experiment with the 5 s retention period block and the other half the 8 s retention period block.

The relatively long retention periods (3–8 s) in the current study were chosen to ensure a large enough dynamic range to manipulate the SOAs in the irregular condition. For example, in Experiment 3, given that the duration of speech stimuli was 350 ms, we manipulated the temporal structure by constraining the SOAs to be within the 400–1100 ms range with a mean of 750 ms, the SOA employed in the temporally regular condition. As a result, the distractor sequence was relatively long (~ 5.6 s) and a long retention period was employed. Differential distraction by speech of varying acoustic detail was also found in a previous study with a retention period longer than the one used in the current study^29^. As the main research interest in the current study was to unravel the difference between temporally regular and irregular distractors, we compared the outcome measures between temporally regular and irregular conditions, holding the retention period constant between conditions within each experiment.

In all experiments, participants were instructed to keep their eyes open and not to speak the target numbers out loud. They were instructed to fixate the fixation cross in the middle of the screen during the encoding (for Experiment 2, 3, and 4) and retention (for Experiment 1 to 4) period whenever a fixation cross was presented.

To check whether temporally regular distractors were perceived as more rhythmic than temporally irregular distractors, we also included a rhythmicity rating for distractor sequences after Experiments 2 and 3. Participants listened to all of the distractor sequences that were presented in the main experiment and rated how rhythmic they found each distractor sequence on a scale from 1 (not rhythmic at all) to 7 (very rhythmic) by clicking the number on the screen with a mouse. The distractor sequences were presented in a randomized order.

Experiments 1, 3, and 4 were implemented using MATLAB (MathWorks, Inc., Natick, USA) and Psychophysics Toolbox^48^. Experiment 2 was implemented as an online study, using Labvanced^49^. Participants used headphones for Experiments 1, 3, and 4, while approximately half of the participants (N = 10) used headphones and the other half (N = 9) used speakers in Experiment 2 (according to self-report). The auditory materials were presented at comfortable listening levels. Details of all experiments are listed in Table 1.

### Analysis

For Experiments 1 to 3, we analyzed the effect of temporal regularity on working memory performance with repeated-measures ANOVAs or paired *t* tests (2-tailed), using the data of individual experiments. To increase the power of the analysis, we also ran a mixed-design ANOVA for the combined data of Experiments 1–4 with temporal regularity as the within-subject factor and experiment as the between-subject factor. Accuracy was operationalized as the proportion correct of the serial recall, which, on the single-trial level, could take on 10 possible values (0–9/9) in Experiments 1 and 2, 9 possible values (0–8/8) in Experiment 3, and 2 binary values (0 = incorrect and 1 = correct) in Experiment 4. To delineate whether the empirical data speak to the alternative versus the null hypothesis, we complemented frequentist statistical analyses with the Bayes Factor (*BF*_*10*_)^50,51^. As an effect size, we report r_equivalent_, which is bound between 0 and 1^52^. r_equivalent_ was derived from Cohen’s d in the paired *t* tests or eta-squared (*η*^2^) from the repeated-measures ANOVAs using the transformation provided in an online tool^53^ (https://www.psychometrica.de/effect_size.html).

For Experiment 1, we ran a paired *t* test with the factor regularity, referring to the temporal delay between the last two distractor tones, which could either agree with the delays between all distractors earlier in the sequence (i.e., 250 ms; denoted regular) or differed systematically (i.e., 125, 187.5, 312.5, or 375 ms; denoted irregular). In addition, we ran repeated-measures ANOVAs to test the effect of the exact delay (in ms) between the last two distractor tones (denoted final distractor onset), as well as the absolute deviation of this delay from a regular distractor (i.e., |250 ms—delay between last two distractor tones|; denoted final distractor deviation).

For Experiments 2 and 3, paired *t* tests were used with the factor regularity, which referred to the temporal structure of the whole distractor sequence. We tested whether temporally regular distractors were perceived as more rhythmic than the temporally irregular distractors in Experiment 2 and 3, respectively, using paired *t* tests with regularity as the factor. In Experiment 3, we recorded participants’ response time in addition to accuracy. Response time (RT) was defined as the time interval between the presentation of the number pad on the screen and participants’ first button press. We first converted response time into speed (1/RT), and then excluded 6% of the slowest (3%) and fastest (3%) trials^54^. We analyzed the effect of temporal regularity on speed by replacing accuracy with speed in the analysis.

For Experiment 4, response time was defined as the time interval between the presentation of the response screen and the button press. We also converted response time into speed (1/RT) and excluded 6% of the slowest (3%) and fastest (3%) trials together with the trials without a response (approximately 3%). We implemented two analysis approaches. First, to examine whether there is an interaction between distractor modality and regularity, we employed repeated-measures ANOVAs on each outcome measure (i.e., accuracy, speed, and confidence) separately, with the factors modality and regularity. As the modality × regularity interaction was not significant, we collapsed across visual and auditory distractors for further analyses.

In addition, we ran trial-wise linear mixed models including trial number and distractor onset delay for all aforementioned analyses. As the patterns of the trial-wise analyses and the analyses on aggregated data converged, the results of the linear mixed models are not presented here.

Second, in Experiment 4, we used signal detection theory^55^ to derive sensitivity and response bias (criterion) separately for Regular and Irregular conditions, respectively, using Eqs. (1) and (2):

1 $${\text{Sensitivity }} = {\text{ z }}\left( {\text{Hit rate}} \right) - {\text{ z }}\left( {\text{False alarm rate}} \right)$$

2 $${\text{Criterion }} = - 0.5 \times \left( {{\text{z }}\left( {\text{Hit rate}} \right) + {\text{ z }}\left( {\text{False alarm rate}} \right)} \right)$$

Hit rates and false alarm rates of 0 or 1 for individual participants were replaced by 1/2 N and 1 − 1/2 N, respectively, where N refers to the number of trials^56^. Since sensitivity and response bias cannot be derived for single trials, we only used paired sample *t* tests to test for effects of distractor regularity.

As participants responded significantly more conservatively in the regular condition (i.e., more positive response bias) in Experiment 4, we further investigated the effect of regularity on the outcome measures. Higher response bias means that participants tend to respond “different” (i.e., “no” response), which suggests that participants may have different confidence ratings depending on whether the target and probe displays matched. Hence, we included the factor match, which classifies whether the target and probe displays were the same (match = 1; correct response = “same”) or different (match = 0; correct response = “different”), into a 2-way repeated-measures ANOVA including the factor distractor regularity. We did not repeat these analyses on sensitivity and response bias as the factor match was taken into account while calculating the two measures (i.e., hit rate: response “same”, match = 1). As in Experiments 1 to 3, we also included effect sizes and Bayes factors to quantify the strength of the evidence towards the alternative hypothesis. Bayes factors indicate the comparison between the likelihood of an alternative hypothesis to that of a null hypothesis^51^. For statistically significant results (i.e., p < 0.05), we used the Bayes factor not to decide whether there is an effect, but rather to estimate the strength of the evidence. More critically, for null results (i.e., p > 0.05), we used the Bayes factor to indicate whether the result is more likely to reflect the absence of evidence or evidence for the absence of an effect. Conventionally, Bayes factors (*BF*_*10*_) > 3 indicate that the observed data are substantially more likely to speak to the alternative hypothesis than the null hypothesis, and vice versa with Bayes factors < 1/3. Bayes factors of 1 indicate that the data do not speak to either the alternative hypothesis or the null hypothesis.

Post-hoc power analyses with 20 and 89 participants were conducted, approximately matching the number of participants in individual experiments and the total number of participants, respectively. With N = 20, alpha = 0.05, and power = 0.80, the minimum effect size needed to reliably observe a significant effect (in a paired samples *t* test) was *r* = 0.31. With N = 89, alpha = 0.05, and power = 0.80, the minimum effect size needed to reliably observe a significant effect was *r* = 0.15. The temporal regularity effects observed in the current study were smaller than these minimum effect sizes. We also compared the effect sizes observed in the current study to previous studies from the literature which found effects of the temporal regularity of distractors on memory recall accuracy^23,24^. The effect sizes in the current study (e.g., *r* = 0.009 in the combined analysis) are considerably smaller than the effect sizes obtained in these studies, which were *r* = 0.42^23^ and *r* = 0.19^24^, respectively. All statistical analyses were conducted in jamovi (version 1.6.23; http://www.jamovi.org).

## Results

### Temporal regularity of distractors does not affect working memory recall accuracy

We tested whether temporally regular versus irregular distractors would differentially affect working memory recall accuracy. Across Experiments 1–4, regular distractors did not interfere more with recall accuracy than irregular distractors (*F*_*1,85*_ = 0.31, *p* = 0.577, *r* = 0.009). The Bayes Factor for this contrast (*BF*_*10*_ = 0.24) provides evidence for the absence of an effect of temporally regular versus irregular distractors on the accurate recall from working memory. The interaction between distractor regularity and experiment was also not significant (*F*_*1,85*_ = 0.51, *p* = 0.680, *r* = 0.020, *BF*_*10*_ = 0.10), suggesting that the absence of the distractor regularity effect was consistent across all four experiments.

**Figure 2:**
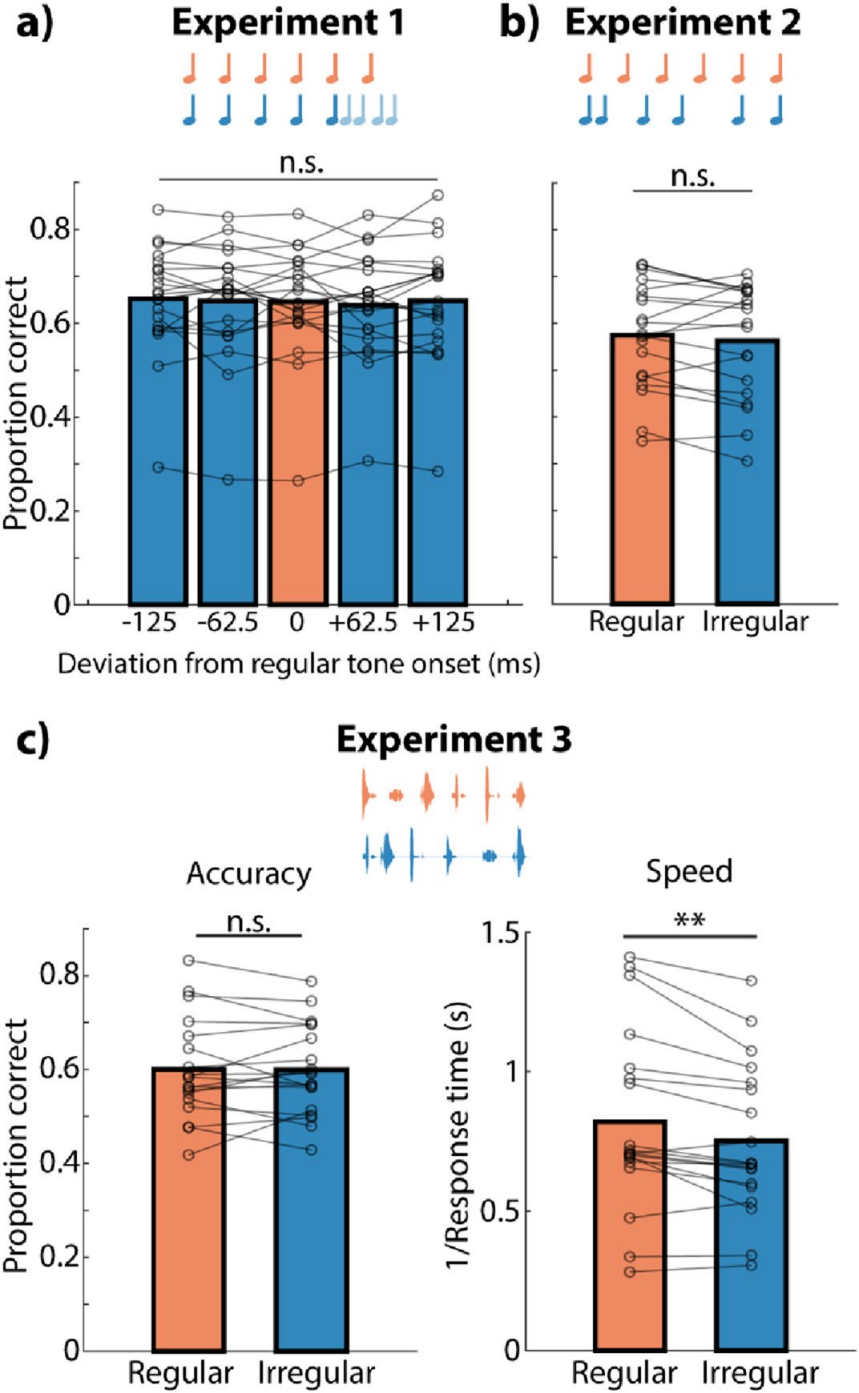
Serial recall performance in Experiment 1 (**a**), Experiment 2 (**b**), and Experiment 3 (**c**). (**a**) Proportion correct for different deviations of the sequence-final distractor tone from temporal regularity in Experiment 1. (**b**) Proportion correct in regular and irregular conditions in Experiment 2. (**c**) Left, Proportion correct in Experiment 3 for regular and irregular distractors. (**c**) Right, Response speed (1/RT). Bars show averages across all participants. Lines show data from individual participants. *n.s.* not significant. **p < 0.01.

No significant effect of distractor regularity (regular versus irregular) was found in the analyses for individual experiments as well. For Experiment 1 (Fig. 2a), whether the delay between the last two distractor tones was the same (regular) or different (irregular) from the 250-ms delays between previous tones in the sequence did not affect task accuracy (*t*_*20*_ = − 0.32, *p* = 0.975, *r* = 0.004, *BF*_*10*_ = 0.23). Also, the exact delay of the final distractor tone (i.e., final distractor onset) and the absolute deviation of the final distractor tone from regular distractor (i.e., final distractor deviation) did not affect working memory recall accuracy (final distractor onset effect, *F*_*4,80*_ = 0.51, *p* = 0.725, *r* = 0.039, *BF*_*10*_ = 0.09; final distractor deviation effect, *F*_*2,80*_ = 0.36, *p* = 0.701, *r* = 0.025, *BF*_*10*_ = 0.17). In Experiment 2, regular distractor tone sequences were not more distracting than irregular sequences (*t*_*18*_ = 1.12, *p* = 0.279, *r* = 0.127, *BF*_*10*_ = 0.41; Fig. 2b). Similarly, no effect on working memory recall accuracy was found for temporally regular versus irregular sequences of spoken numbers in Experiment 3 (*t*_*18*_ = 0.07, *p* = 0.945, *r* = 0.008, *BF*_*10*_ = 0.24; Fig. 2c, left panel). Importantly, however, temporally regular distractors were perceived as more rhythmic than irregular distractors in both Experiment 2 (*t*_*17*_ = 2.32, *p* = 0.033, *r* = 0.264, *BF*_*10*_ = 2.00) and Experiment 3 (*t*_*18*_ = 8.02, *p* < 10^−5^, *r* = 0.677, *BF*_*10*_ > 10^5^).

**Figure 3:**
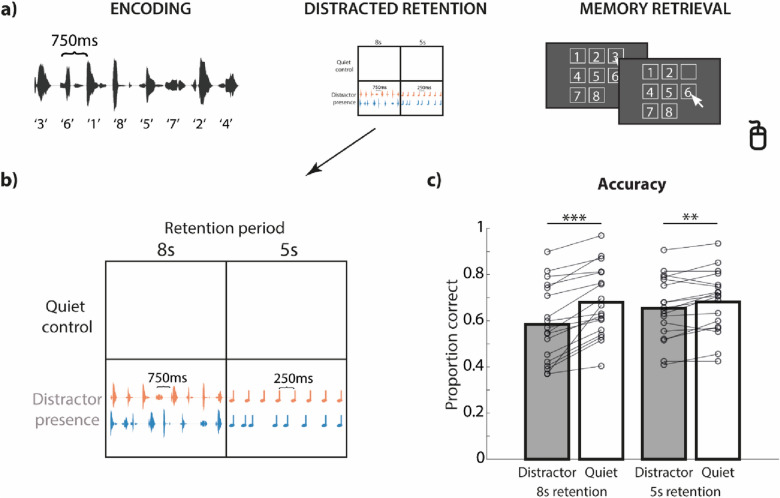
(**a**) Illustration of the trial structure in the control experiment, which was largely identical to the trial structure in Experiment 3. (**b**) Four conditions were implemented to cross the factors distractor (present vs absent) and duration of retention period (5 s vs 8 s). (**c**) Proportion correct scores in each condition in the control experiment. Lines show data from individual participants. **p < 0.01, ***p < 0.001.

To ensure that the lack of a temporal regularity effect cannot be attributed to a weak distraction effect in general, we additionally compared memory recall accuracy in the distractor-presence condition with a distractor-absence condition (i.e., quiet control condition) in a control experiment (Fig. 3). The distractor presence × retention duration interaction was significant (*F*_*1,17*_ = 29.10, *p* < 0.001, *r* = 0.114, *BF*_*10*_ = 13.36), suggesting that the disruptive effect by different distractors varied. Post-hoc tests revealed that participants performed worse when tone distractors (*t*_*17*_ = − 3.30, *p* = 0.008, *r* = 0.336, *BF*_*10*_ = 6.68) and when spoken number distractors (*t*_*17*_ = − 6.17, *p* < 0.001, *r* = 0.588, *BF*_*10*_ = 2174.82) were presented, compared with the quiet control with the same retention period duration. Participants’ memory recall accuracy did not differ for different retention period durations in the quiet control condition (*t*_*17*_ = − 0.10, *p* = 0.921, *r* = 0.012, *BF*_*10*_ = 0.24). They performed worse with speech distractors in the 8 s retention period block than with tone distractors in the 5 s retention period block (*t*_*17*_ = − 4.68, *p* < 0.001, *r* = 0.483, *BF*_*10*_ = 142.80). In sum, results of the control experiment demonstrated that the distractors used in the main experiments were indeed distracting.

**Figure 4:**
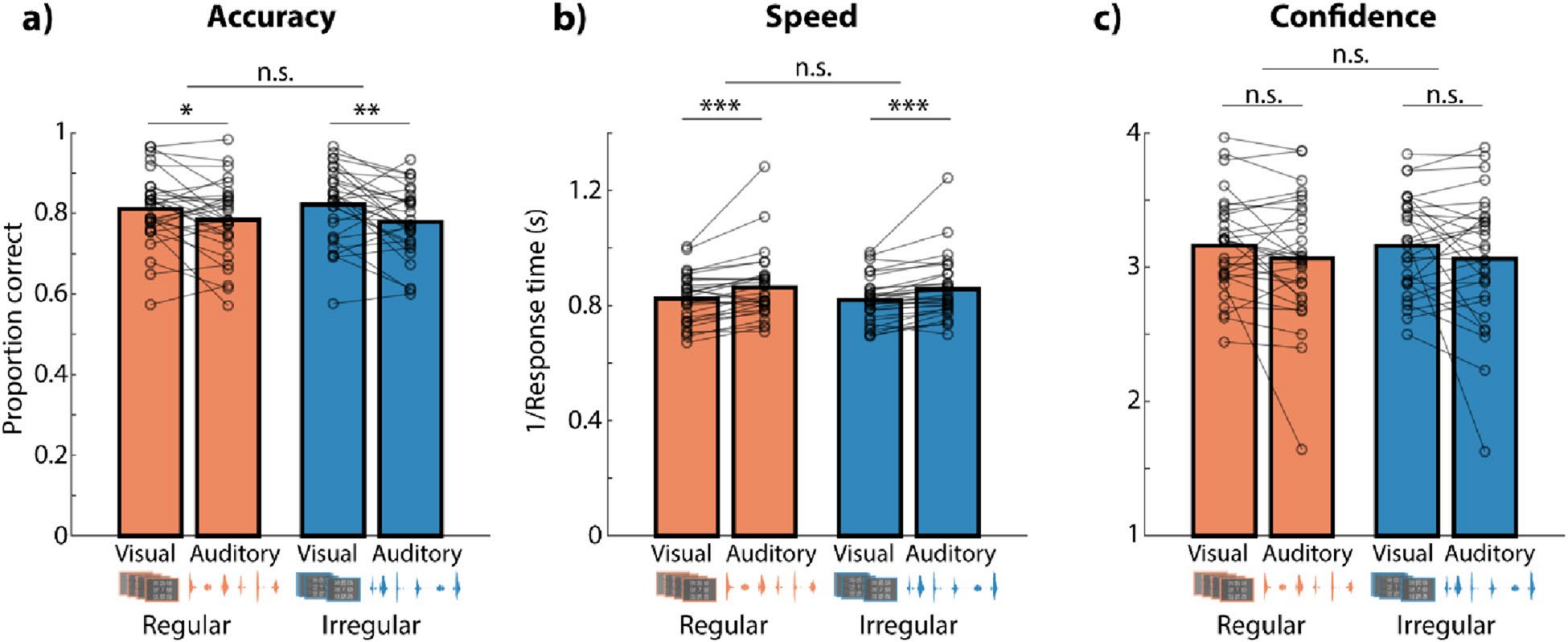
Bars show average accuracy (**a**), speed (**b**), and confidence rating (**c**) for distraction in different modalities (visual vs. auditory) and for regular vs. irregular distractors in Experiment 4. Lines show data from individual participants. *n.s.* not significant, *p < 0.05, **p < 0.01, ***p < 0.001.

Figure 4 shows the effects of distractor modality and regularity on different outcome measures in Experiment 4. For auditory compared with visual distractors, accuracy was lower (*F*_*1,29*_ = 9.96, *p* = 0.004, *r* = 0.193, *BF*_*10*_ = 203.82) and responses were faster (*F*_*1,29*_ = 15.92, *p* < 0.001, *r* = 0.193, *BF*_*10*_ > 10^5^), but confidence did not differ significantly (*F*_*1,29*_ = 2.45, *p* = 0.129, *r* = 0.115, *BF*_*10*_ = 2.66). The main effect of regularity and the modality × regularity interaction were not significant for any of the measures (all *F* < 1.5, all *p* > 0.25).

### Temporal regularity of distractors affects response behavior

We tested whether the temporal regularity of distractors influences secondary performance metrics in Experiments 3 and 4. As we additionally recorded response time in Experiment 3, we also investigated the effect of temporal regularity on the speed of the first manual response (i.e., first click on a number on the response screen). Participants responded significantly faster when the distractor sequence during retention was temporally regular (Fig. 2c, right panel; *t*_*18*_ = 3.61, *p* = 0.002, *r* = 0.383, *BF*_*10*_ = 20.3).

**Figure 5:**
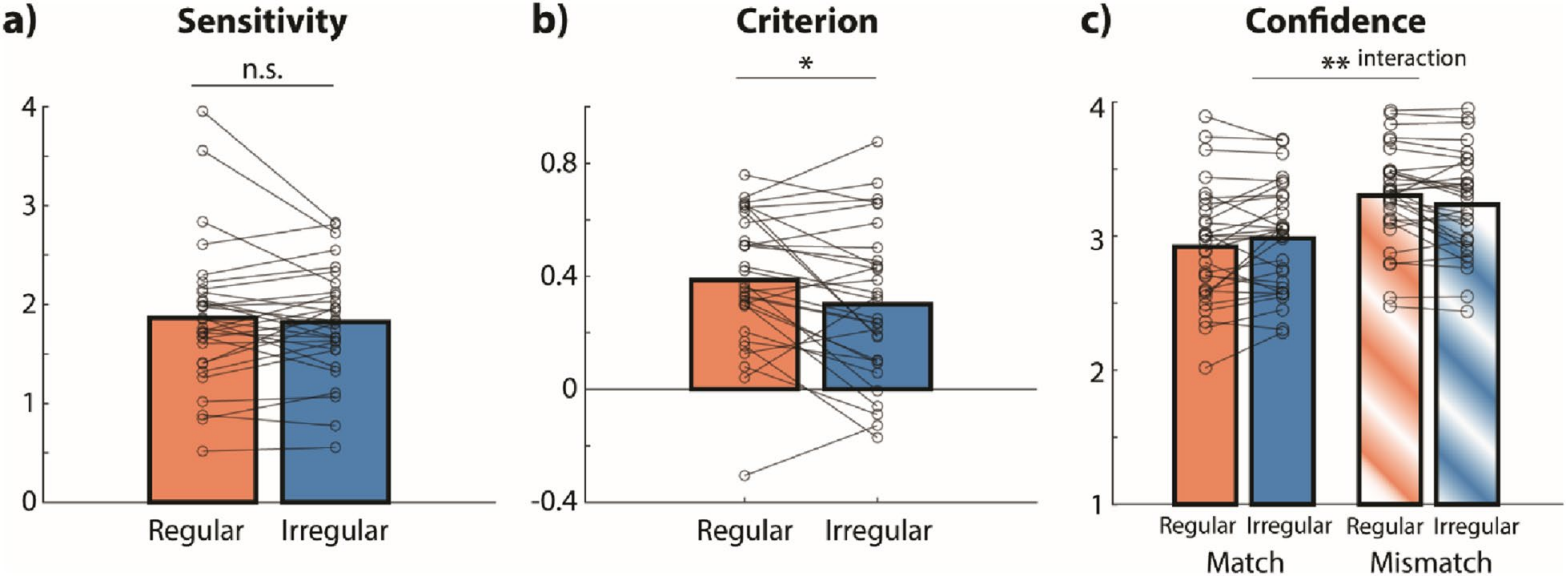
Sensitivity (**a**) and criterion (**b**) for temporally regular versus irregular distractors in Experiment 4. Bars shows means across all participants (N = 30). Lines show data from individual participants. (**c**) Interactive effect of temporal regularity and match, which refers to whether the memory probe matched with the encoding display, on confidence. *n.s.* not significant. *p < 0.05, **p < 0.01.

In Experiment 4, we also probed into the effect of distractor regularity using outcome measures derived from signal detection theory. Figure 5a,b show sensitivity and response bias (criterion), respectively, in regular and irregular conditions. We collapsed across modalities as no interaction between distractor modality and regularity was found in the previous analyses. Participants’ sensitivity was not modulated by the temporal regularity of the distractor (*t*_*29*_ = 0.62, *p* = 0.542, *r* = 0.056, *BF*_*10*_ = 0.23). However, they responded more conservatively (i.e., higher tendency to respond “probe differs from encoding display”) when the distractor was temporally regular versus irregular (*t*_*29*_ = 2.50, *p* = 0.019, *r* = 0.222, *BF*_*10*_ = 2.67).

To follow-up on the effect of distractor regularity on response bias, we split up the metacognitive measure confidence in Experiment 4 for trials in which the memory probe matched versus mismatched with the encoding display. We thus used a repeated-measures ANOVA with the factors match and regularity, which revealed a significant match × regularity interaction on confidence with moderate evidence towards the alternative hypothesis (*F*_*1,29*_ = 9.03, *p* = 0.005, *r* = 0.075, *BF*_*10*_ = 3.18). Figure 5c shows that participants were more confident in trials with regular distractors when the probe and target numbers did not match, and vice versa in the match condition.

## Discussion

In the present study, we systematically manipulated different aspects of temporal regularity in distractor sequences and probed their impact on working memory. Distractor regularity did not modulate memory recall (Experiments 1–3) or recognition accuracy (Experiment 4), demonstrating that the absence of this effect is robust and generalizes to different variations of working memory paradigms and operationalizations of temporal regularity. Nevertheless, regular versus irregular distractors had an influence on response behavior, as reflected in response speed (Experiment 3), response bias, and confidence (Experiment 4). Our findings show that although temporal regularity of distractors does not inevitably affect primary performance metrics (recall accuracy), (ir)regularity of distractors does not go unnoticed and affects secondary performance metrics, which are often neglected in this field of research. A comprehensive understanding of auditory distraction requires that existing models of attention include secondary performance measures beyond recall accuracy.

### No effect of temporal regularity of distractors on memory recall accuracy

The null results found in the current study may seem, at first glance, at odds with a previous study where the temporal regularity of distractors influenced target detection performance^22^. A crucial difference, however, is that all experiments in the present study separated the distractor in time from the target stream, which eliminated potential masking or interference effects during the encoding period. Thus, the present study tested the interfering effect of temporally regular versus irregular distractors on memory retention only, whereas higher interference for temporally regular distractors in the study by Makov and Zion Golumbic^22^ might reflect interference of target encoding.

Previous studies found inconsistent evidence for memory interference by temporally regular versus irregular distractors^23,24^. Jones and Macken^23^ suggested that a temporally irregular distractor sequence implements a changing-state sequence, which increases the disruption of working memory. In contrast, Parmentier and Beaman^24^ argued that irregular distractor sequences exhibit less distraction since distractors that are closer in time in an irregular stream might be grouped together, resulting in fewer transitions between units^57^. Both accounts approached temporal regularity of distractors as an attribute that influenced the degree of distraction by means of sequence segmentation. The current study could not support the speculations from either study as we found that regular versus irregular distractors did not affect memory retrieval at all, neither in the analyses on individual experiments nor the combined analysis. Of note, both studies mentioned had distractors presented throughout the entire encoding and maintenance period. It is thus possible that the temporal regularity of distractors has a bigger impact on memory encoding than on maintenance. Also, the current study only presented the distractor sequences in a portion of the retention period. In theory, it is possible that a longer sequence of distractors is needed to detect a temporal regularity effect of distractors on memory maintenance.

The current study revealed a null effect of temporal regularity of distractors on working memory recall accuracy by ensuring that the absence of an effect was not specific to certain experimental manipulations. Across the four experiments, we included visual or auditory targets, different distractor stimuli (e.g., pure tones or spoken numbers), as well as different manipulations of temporal regularity (e.g., violation or build-up of temporal regularity). We also complemented frequentist statistical analysis with Bayesian statistics to reveal whether non-significant results were more likely to arise from a true null effect (BF < 1) or were indifferent to null versus alternative hypotheses (BF = 1)^51^. The Bayes factors smaller than 0.33 across different analyses (e.g., BF_10_ = 0.24 in the combined analysis of Experiments 1–4) suggest that temporal regularity of distractors during memory retention does not affect memory recall accuracy.

Here, we discuss three possible explanations for why the temporal regularity of distractors did not influence working memory performance. First, the influence of temporal regularity of distractors on memory retention may be frequency-specific. In attention research, rhythmic stimuli have been shown to modulate participants’ performance maximally at 2–3 Hz^58^, which falls into the range of the hypothesized resonance frequency of the attention network^59^. It is possible that a resonance frequency also exists for the vulnerability to distraction. In a recent study, we found that the vulnerability to speech distractors fluctuates at around 2.5 Hz^20^. It might thus be that the frequencies of temporally regular distractors in the current study were either too slow (1.33 Hz in Experiment 3 and 4) or too fast (4 Hz for Experiment 1 and 2) to exert an influence on the eventual memory recall that would differ from temporally irregular distractors.

Second, it is possible that the irregular temporal structure we used in the current study, albeit being physically aperiodic, may be perceived as rhythmic by the participant. However, we do not consider it likely due to the results of the rhythmicity rating in Experiment 2 and 3. In the current study, we defined temporal (ir)regularity in a strict manner by isochronous versus non-isochronous temporal structures. Stimuli with non-isochronous temporal structure, such as metrical musical rhythm or jittered SOA, may also be perceived as rhythmic and hence influence behavior similarly to those with isochronous temporal structure^60–62^. As we also included rhythmicity ratings in Experiments 2 and 3, we additionally compared the perceived rhythmicity between temporally regular and irregular distractors. Temporally regular distractors were indeed perceived as more rhythmic than irregular distractors. It is thus not likely that the absence of the effect arises from perceived rhythmicity of temporally irregular distractors in the current study.

Third, while previous studies demonstrated that neural or behavioral responses could be entrained by, i.e., temporally aligned to, temporally regular target stimuli^63^, whether temporally regular distractors also exert similar influence remained unclear. The absence of effect in the current study agrees with the view that entrainment requires attention^64,65^. In contrast with how we can better attend to the target stimuli presented at the expected time point, we are not more or less distracted by distractors presented at the expected time point compared with distractors presented at a random time point.

### Secondary performance metrics are sensitive to temporal regularity of distractors

The results in Experiments 3 and 4 revealed that the temporal regularity of distractors posed an influence on participants’ response behavior. Temporal structures of stimuli, such as higher cueing frequency^66^ or periodicity^32,67^, were found to have an impact on response speed. Consistently, in Experiment 3, participants responded faster after being exposed to regular distractors compared to irregular distractors. The facilitatory effect of temporal regularity on response speed might suggest that the readiness to respond may be modulated by the temporal regularity in distractors. A previous study using button presses as responses did not find a speed difference between rhythmic versus no distractors^68^. The current study differed from this study in terms of the operationalization of temporal regularity (i.e., identical SOA versus repeating temporal structure), response type (i.e., by mouse versus by button presses), and control condition (i.e., irregular temporal structure versus quiet). In agreement with the present study, a recent study showed that temporal regularity of target stimuli led to motor preparation when a mouse was used as response device^45^. Hence, it is possible that the periodicity embedded in regular distractors in Experiment 3 facilitates response speed through increased motor preparation.

No such speed difference between regular and irregular distractors was found in Experiment 4, which may be attributed to the difference in response type or task between the two experiments. Participants knew the first number to select from the response screen already during the retention period in Experiment 3, while they only knew the correct button press when the probe number was displayed on the response screen in Experiment 4. As a result, participants had ample time to prepare for the motor response in Experiment 3, but not in Experiment 4.

In Experiment 4, temporal regularity of distractors did not affect the accuracy of working memory but rather secondary performance metrics of participants. We found that response bias was more positive for regular distractors, indicating a stronger tendency to report a mismatch between encoding and probe displays and to respond “different”. Since this effect was unexpected, we can here only speculate about the underlying mechanisms. Response bias, and associated confidence ratings, were previously found to be subject to various factors such as the probability of a signal^69,70^. While we balanced the trial number of match and mismatch trials in Experiment 4, only 1 out of 8 numbers would match with the probe number in a match trial. This low probability of a match (i.e., signal) within a trial may contribute to the generally conservative behavior of participants. With temporally regular distractors, participants may lean more towards their preferred response behavior, which eventually results in more conservative (Experiment 4) and faster responses (Experiment 3). Furthermore, participants’ higher confidence when correctly responding “different” for trials with a regular versus irregular distractor is in line with this interpretation.

The distraction-effects on secondary performance metrics (speed and response bias) and metacognition (confidence) found in the current study speaks to the necessity to acknowledge these measures when the goal is to derive a comprehensive understanding of auditory distraction^71^. The role of distraction in the metacognitive evaluation of working memory performance has only been considered recently^42,71^. Beaman et al.^71^ found that distraction during encoding and retrieval interfered with the resolution of metacognitive monitoring when compared with quiet control. Kattner and Bryce^42^ showed that confidence diminished with a higher degree of distraction during encoding and retention. Our study demonstrated that distractors presented solely in the retention period also pose an influence on metacognitive evaluation and response behavior, suggesting that the effect of distraction may be pervasive on cognition but also on metacognition.

## Conclusion

The current study demonstrates that temporal regularity in the distractor stream during the retention period influenced response behavior in working memory tasks. While distractor regularity in time did not affect the precision of the memory representation, it modulated the response behavior and metacognitive evaluation of memory recall or recognition, reflected by response speed, bias, and confidence. The results of the current study set the stage for future research by showing the impact of temporal regularity in task-irrelevant stimuli on the often-neglected secondary performance metrics of goal-directed behavior. Theoretically, the current study highlights the importance to yield a comprehensive understanding of how auditory distraction reaches awareness, and ultimately impacts task-relevant cognitive processes, by including these secondary performance metrics in existing models of attention.

## Data Availability

Data tables to reproduce all analyses reported in the present article are available online (https://osf.io/m3pj2/). Raw data are available from the corresponding authors upon request.
